# Skeletal muscle fuel utilisation during exercise: A historical account of the Scandinavian involvement in the ‘Zuntz–Chauveau controversy’

**DOI:** 10.1113/EP092156

**Published:** 2024-10-18

**Authors:** Ronan M. G. Berg

**Affiliations:** ^1^ Centre for Physical Activity Research Copenhagen University Hospital – Rigshospitalet Copenhagen Denmark; ^2^ Department of Clinical Physiology and Nuclear Medicine Copenhagen University Hospital – Rigshospitalet Copenhagen Denmark; ^3^ Department of Biomedical Sciences, Faculty of Health and Medical Sciences University of Copenhagen Copenhagen Denmark; ^4^ Neurovascular Research Laboratory, Faculty of Life Sciences and Education University of South Wales Pontypridd UK

1

As I have previously recounted, the collective research efforts of Erik Hohwü Christensen (1904–1996), Marius Nielsen (1903–2000) and Erling Asmussen (1907–1991), known as The Three Musketeers of Scandinavian exercise physiology, significantly advanced the understanding of the adaptive resetting of homeostasis across organ systems during exercise (Berg, 2024). Their collaborative studies, conducted mainly between 1936 and 1941, primarily focused on cardiovascular, respiratory and thermoregulatory functions. At first glance, one might wonder why they did not also focus on skeletal muscle metabolism within this framework; indeed, is the very *raison d'être* of these homeostatic adaptations not to ensure adequate substrate delivery to the contracting skeletal muscle? However, it turns out that this was indeed a primary research focus for Christensen and his colleague Ove Hansen (1907–1990) as early as 1931–1932. Here, I describe how their studies added to the decades‐long so‐called ‘Zuntz–Chauveau controversy’, but how unforeseen events seemingly stalled this line of research for years, until revived by Christensen's students at the Royal Gymnastic Central Institute (GCI) in Stockholm.

The ‘Zuntz–Chauveau controversy’ centred on whether carbohydrates serve as the exclusive substrate for skeletal muscle metabolism or not, particularly during exercise (Asmussen, 1971). The French veterinarian and Professor of Comparative Pathology Jean‐Baptiste Auguste Chauveau (1827–1917) is credited as the first to propose that glucose is the sole metabolic substrate for muscle contraction, as his experiments showed that the levator muscle of the lower lip in horses increases glucose uptake during chewing (Chauveau & Kaufman, 1887). This supported his subsequent demonstration that the respiratory exchange ratio (RER) in humans increases from 0.70 at rest to 0.97 after hours of stair‐walking (Chauveau, 1896). From the late 1880s, the contemporary Berlin‐based physiologist Nathan Zuntz (1847–1920) also measured a rise in RER in exercising horses and humans. However, when Zuntz later reviewed his almost 30 years of work (Zuntz, 1911), he reasoned that since RER never reaches unity during exercise, both carbohydrates and fats must be oxidised by contracting skeletal muscle. Zuntz furthermore argued that the relative contribution of each substrate depends on the intensity and duration of the exercise.

Things (literally) started heating up when the rising star of the time, Archibald V. Hill (1886–1977), disputed Zuntz's theory. Hill had shown that heat production in contracting isolated frog muscle remained constant regardless of oxygen availability, but that additional heat was generated when oxygen was present during the recovery phase (Hill, 1910, 1913). Hill attributed this phenomenon to the formation of lactate, which he believed to be the primary substrate for muscle contraction. He proposed that during recovery, oxygen was utilised to convert lactate back into its parent carbohydrate molecule (Hill, 1913), later identified as glycogen. In parallel, Francis G. Benedict (1870–1957) and Edward P. Cathcart (1877–1954) studied a professional cyclist; using a mouthpiece and closed‐circuit system for intermittent collection of expired air during exercise, they found that RER increased during exercise, regardless of whether this was preceded by a high carbohydrate or high‐fat diet (Benedict & Cathcart, 1913). However, RER did not reach unity, and they thus concluded that there was compelling evidence to support increased carbohydrate utilisation during exercise, but stressed that their findings did not necessarily imply exclusive reliance on carbohydrate metabolism for skeletal muscle contraction (Benedict & Cathcart, 1913).

August Krogh (1874–1949) and Johannes Lindhard (1870–1947) at the University of Copenhagen, closely followed the ongoing debate regarding skeletal muscle fuel utilisation whilst conducting their studies on acute exercise in humans. Back in 1908, August Krogh and his wife, Marie Krogh (1874–1974), had obtained RER measurements in Innuits on an expedition to Greenland (Krogh & Krogh, 1913). Since then, August Krogh has optimised his RER measurements by constructing an elaborate respiration chamber to achieve unprecedented precision for the measurement of respiratory gas fractions with an average difference between double determinations of 0.001 percentage points or less (!) (Krogh, 1920). As August Krogh and Lindhard were very critical of the measurements obtained by the intermittent air collection technique employed by Benedict and Cathcart, they devised their own extensive experimental set‐up, in which they subjected six individuals—including themselves—to a controlled three‐day high carbohydrate or high‐fat diet. The high‐carbohydrate diet consisted of bread, cakes, apples, potatoes and green peas, whilst the high‐fat diet included bacon, cream, butter, eggs and cabbage. The latter proved particularly cumbersome, often causing gastrointestinal discomfort and disrupted sleep (Krogh & Lindhard, 1920). Following the dietary regimens, the participants underwent steady‐state measurements of RER during exercise at a light‐to‐moderate intensity. August Krogh and Lindhard published a 74‐page paper in 1920 with more than 200 RER determinations, showing a consistent increase in RER during exercise, especially pronounced after the high‐carbohydrate diet, but RER always remained below unity (Krogh & Lindhard, 1920). They therefore affirmed that whilst carbohydrates are the primary substrate for muscular work, fat utilisation is also a source of energy, depending on diet, exercise intensity and exercise duration.

In the early 1930s, a comprehensive research programme into the physiology of strenuous exercise was undertaken at August Krogh's Zoophysiological Laboratory and Lindhard's Laboratory for Gymnastics Theory at the Rockefeller Institute, partly funded by the League of Nations Health Organisation (Christensen et al., 1934). Based on one of Christensen's earlier investigations, showing hypoglycaemia as a cause of exhaustion during prolonged strenuous exercise (Christensen, 1931), and inspired by August Krogh and Lindhard's study from a decade prior, Christensen and Hansen pushed the boundaries further by incorporating even more strenuous and extended exercise sessions lasting up to 4 h. This was much to the frustration of the resident professors at the Rockefeller Institute, as these sessions were often initiated between 6 and 7 a.m. (Asmussen, 1987). As in his previous studies, and probably inspired by the work of Arlie Bock (1888–1984) and co‐workers from the Harvard Fatigue Laboratory who had also measured RER during exercise (Bock et al., 1928), Christensen's work focused on measurements obtained at steady state, and in contrast to August Krogh and Lindhard's earlier studies, the measurements were obtained on highly fit individuals, which typically included himself, Hansen and other colleagues.

In their experiments, Christensen and Hansen implemented much more extensive dietary interventions than August Krogh and Lindhard, spanning week‐long periods of either high‐carbohydrate or high‐fat diets. The dietary regimen of three daily high‐fat meals, typically comprising fatty pork, whipped cream and mayonnaise, proved to be particularly arduous. Even with the assistance of a colleague's wife, a household assistant who did her best to make the meals palatable, it was challenging to adhere to the diet. To cope with the dietary challenges, Christensen and Hansen often had to flush down these meals with a snaps or two (Asmussen, 1987).

Christensen and Hansen found that RER served as a valid indicator of the relative proportions of fat and carbohydrate utilisation during exercise, particularly at submaximal levels, but not at maximal exertion (Christensen & Hansen, 1939a). Notably, RER was only reliable once a steady state had been established, typically requiring 10–15 min of exercise at a given intensity (Christensen & Hansen, 1939a). They observed that RER increased with exercise intensity and that this increase was especially pronounced after a high‐carbohydrate diet (Christensen & Hansen, 1939b, 1939c). Furthermore, well‐trained individuals exhibited lower RER values at the same external workload compared to untrained individuals (Christensen & Hansen, 1939d). During prolonged exercise, they noted a decrease in RER (Christensen & Hansen, 1939b, 1939c), indicating a metabolic shift towards increased fat utilisation, and they also found that endurance could be enhanced by oral glucose ingestion during exercise (Christensen & Hansen, 1939e). They interpreted their findings to suggest that whilst carbohydrates played a predominant role in muscle energy turnover, fat was an alternative substrate which could serve to spare the body's glycogen stores, thereby influencing endurance. However, the mechanisms governing this regulation remained to be determined, and there was still no direct evidence of glycogen depletion in skeletal muscle as a mechanism of exhaustion since skeletal muscle glycogen could not be measured directly.

As Christensen and Hansen were completing their experiments in August Krogh's Zoophysiological Laboratory in 1932, Hansen's health took a dramatic turn for the worse (Asmussen, 1987). He began experiencing personality changes, progressive depressive symptoms and compulsions. Eventually, his condition deteriorated to the point where he required extended sick leave. Hansen had spent hours after each experiment in the basement under the Zoophysiological Laboratory where the gas analyser was located. The handling of mercury at the laboratory was notoriously reckless at this time, and it was often spilled and left in pools on tables and both on and underneath the wooden floorboards (Schmidt‐Nielsen, 1995). Later, many of Hansen's colleagues speculated that his symptoms were caused by the prolonged exposure to mercury vapours. Indeed, both Christensen and Nielsen later suffered from acute mercury poisoning with pulmonary oedema during an overnight experiment in the laboratory's hypobaric chamber. Had it not been for their concerned wives who insisted on staying by to check up on them, this could have caused an untimely end to the tales of The Three Musketeers (Asmussen, 1987)! Whilst the overnight experiments were never completed, Christensen and Nielsen managed to maximise the scientific gain from their somewhat dramatic experience, as they subsequently published it as a case study on mercury poisoning in *Nature* with the assistance of Marie Krogh (Christensen et al., 1937).

It took five years before Christensen and Hansen got to write up their findings on skeletal muscle fuel utilisation, such that they could finally be published in five back‐to‐back papers (Christensen & Hansen, 1939a, 1939b, 1939c, 1939d, 1939e). However, they never had the opportunity to follow up on their research, as Christensen had become deeply involved in studying respiratory, cardiovascular and thermoregulatory functions with his fellow Musketeers in the meantime (Berg, 2024). Soon after, they parted ways as Christensen accepted the chair of Physiology of Bodily Exercises and Hygiene at the newly established Department of Physiology at GCI in 1941.

Moving to GCI was a dramatic change for Christensen. He was not met with any immediate welcoming warmth from the management (Åstrand, 1991), and soldiers from the Swedish Air Force were stationed on the premises where he was supposed to establish a laboratory (Schantz, 2009, 2015). This led to an immediate desire to return to Copenhagen, but after the German occupation of Denmark, he ultimately decided to stay in Stockholm (Åstrand, 1991). It took a few years before he could finally move into a new laboratory with two former students as assistants, two refugees from Estonia and a clerk (Åstrand, 1991). The opportunity to perform advanced research was very limited at first, but he gradually created an attractive and internationally renowned research environment, within which he helped break the tradition of theoretically based so‐called Ling gymnastics founded at GCI to instead promote exercise science as an experimental discipline (Schantz, 2009). In line with his formative years as one of The Three Musketeers, he proved himself to be a true swordsman of physiology like so many others before him (Bailey et al., 2023). Despite Christensen's interest in skeletal muscle fuel utilisation in his youth, the research at his laboratory focused on almost every other aspect of exercise physiology for many years (Åstrand, 1991). However, things took a change when Bengt Saltin (1935–2014), a medical student at the Karolinska Institute, joined Christensen's team at GCI nearly two decades after Christensen had accepted the chair.

Saltin's early efforts focused on studying the mechanisms of exhaustion in both untrained and trained individuals, with the latter group mainly comprising champion cross‐country skiers, primarily investigating aerobic capacity and thermoregulation (Saltin, 1964). In his studies, he also measured RER during exercise, and like Christensen and Hansen, he observed that RER increased during several hours of prolonged submaximal strenuous exercise, and then decreased near exhaustion (Saltin, 1964; Saltin & Stenberg, 1964). Whilst this suggested glycogen depletion as a mechanism of exhaustion, it remained speculative, such that all Saltin could do in his doctoral thesis from 1964 was to exclaim, ‛it is impossible to make an exact determination of the glycogen combustion’ (!) (Saltin, 1964). However, this was soon to change, as it turned out that two physicians, Jonas Bergström (1929–2001) and Erik Hultman (1925–2011), working just around the corner at St Erik's Hospital in Stockholm, had developed a muscle biopsy needle technique that could be used for quantitative glycogen measurements in humans.

Initially, Bergström and Hultman investigated skeletal muscle glycogen stores in patients with diabetes mellitus and after surgical stress (Bergström et al., 1965, 1963). However, their research soon caught the attention of Saltin and his colleagues at GCI, as they started studying glycogen depletion in working skeletal muscle. Not only did they publish a paper in *Nature* on the involvement of a local humoral factor in glycogen synthesis after exhaustive exercise (Bergström & Hultman, 1966a), reminiscent of the ‘work factor’ previously proposed by the Three Musketeers (Berg, 2024), but they also specifically reported on the glycogen depletion rate in the vastus lateralis muscles of healthy young men during moderate‐intensity cycle ergometer exercise (Bergström & Hultman, 1966b). As they concurrently measured negligible glucose exchange across the leg, they could conclude that glycogen was indeed the main energy source during this type of exercise (Bergström & Hultman, 1966b). Building on Christensen and Hansen's findings from two decades prior, they also used their muscle biopsy‐based technique to show that glycogen was specifically depleted in contracting muscle and that glucose infusion lessened the glycogen depletion rate (Bergström & Hultman, 1967). Clearly, Saltin was eager to implement their muscle biopsy methodology at GCI, and he soon joined forces with them to follow up on Christensen and Hansen's previous studies.

In their first collaborative study, Bergström, Hultman and Saltin recruited nine healthy volunteers to investigate the depletion of glycogen stores through strenuous exercise to exhaustion (Bergström et al., 1967). Participants underwent a 3‐day dietary intervention involving either a high‐carbohydrate or a combined high‐fat high‐protein diet. Subsequently, muscle biopsies were taken to assess glycogen levels, and the participants then engaged in exercise at a relative workload of 75% of ${\dot{V}}_{O_{2}\max}$ until exhaustion. As expected, the high‐carbohydrate diet led to the highest muscle glycogen levels, and the time to exhaustion correlated closely with the decrease in muscle glycogen content. In another study, participants also worked to complete exhaustion on a cycle ergometer at around 75% of ${\dot{V}}_{O_{2}\max}$, but this time comparing 10 well‐trained to 10 untrained individuals (Hermansen et al., 1967). Glycogen content was determined at several sampling times from the onset of exercise to exhaustion. Carbohydrate combustion corresponded closely to the glycogen depletion rate, which was similar in trained and untrained individuals, and at exhaustion the glycogen content was close to zero in both groups. In a study of long‐distance runners, this glycogen depletion was later found to be fibre‐type specific (Costill et al., 1973). Saltin reasoned that exhaustion likely ensues because free fatty acids and glucose from blood are insufficient energy sources for ATP production when glycogen is depleted, thus rendering the specific fibres unable to accommodate the demand for tension development at the given workload (Saltin, 1975). So, finally, and in line with Christensen and Hansen's previous findings, it could be concluded that glycogen is the main – but not the only – skeletal muscle fuel during strenuous exercise, and its depletion is a critical mechanism of exhaustion. Furthermore, it seemed that greater aerobic fitness enhanced the metabolic capacity for fat utilisation during exercise, such that skeletal muscle glycogen was preserved to enhance endurance.

Curiously, whilst much of the work was going on at GCI in the 1960s, Christensen had decided to embark on a sabbatical, working for the International Labour Organization as an advisor in industrial physiology for the Indian government (Asmussen, 1987). Afterwards, he spent a few years in Copenhagen before he returned to GCI until his retirement. Shortly before his retirement, a symposium entitled ‘Muscle Metabolism During Exercise’ was held at the Karolinska Institute in 1970 with him as the honorary guest. Asmussen gave the introductory talk on the history of research within skeletal muscle energetics, and credited Christensen with resolving the ‘Zuntz–Chauveau controversy’ (Asmussen, 1971). As also highlighted in a discursive monograph in Danish with the translated title *The Special Theory of Bodily Exercises*, which Asmussen and Christensen wrote together during those years (Asmussen & Christensen, 1973), the line of studies going back to August Krogh, Lindhard, The Three Musketeers and their companions all the way up to the studies of Bergström, Hultman and Saltin were critically reviewed. Together, these studies made it clear that skeletal muscle fuel utilisation, akin to respiratory, cardiovascular and thermoregulatory functions, is a tightly regulated process that is reset according to exercise intensity to preserve energy homeostasis. Although this concept was conceived more than a century ago, the factors that mediate and regulate skeletal muscle fuel utilisation remain a major area within exercise physiology and endocrinology to this day (Hargreaves & Spriet, 2020; Smith et al., 2023), studied by generations of Musketeerian swordsmen and swordswomen of physiology, both inside and outside Scandinavia.

## AUTHOR CONTRIBUTIONS

Ronan M. G. Berg conceived and wrote the article and wrote the first draft and is accountable for all aspects of the work in ensuring that questions related to the accuracy or integrity of any part of the work are appropriately investigated and resolved.

## CONFLICT OF INTEREST

The author declares no conflicts of interest.

## FUNDING INFORMATION

The Centre for Physical Activity Research (CFAS) is supported by TrygFonden (grants ID 101390 and ID 20045). The funders had no role in study design, data collection and analysis, decision to publish, or preparation of the manuscript.
